# The Acute Effects of Internal, External, and Golf-Specific Attentional Focus Cues on Isometric Trunk Strength in Youth Golfers

**DOI:** 10.3390/jfmk10040435

**Published:** 2025-11-07

**Authors:** Raouf Hammami, Achraf Hammami, Yassine Negra, Rimeh Staff, Jason Moran, Roland van den Tillaar

**Affiliations:** 1 Higher Institute of Sport and Physical Education of Ksar-Said, University of Manouba, University Campus, Manouba 2010, Tunisia; raouf.cnmss@gmail.com (R.H.); hamami.achref85@gmail.com (A.H.); yassinenegra@hotmail.fr (Y.N.); rimeh.sattaf@hotmail.com (R.S.); 2 Tunisian Research Laboratory “Sports Performance Optimization” (CNMSS-LR09SEP01), National Center of Medicine and Science in Sports (CNMSS), Tunis 1004, Tunisia; 3 Research Laboratory (LR23JS01) “Sport Performance, Health & Society”, Higher Institute of Sport and Physical Education of Ksar-Said, Manouba University, Tunis 1003, Tunisia; 4 School of Sport, Rehabilitation and Exercise Sciences, University of Essex, Colchester CO4 3WA, UK; jmorana@essex.ac.uk; 5 Department of Sport Sciences and Physical Education, Nord University, 937601 Levanger, Norway

**Keywords:** attentional focus, internal cues, external cues, sport-specific cues, trunk strength, back extensors, youth golfers, motor control, sports performance, coaching strategies

## Abstract

**Background**: Attentional focus strategies, including internal, external, and sport-specific cues, can influence muscle strength by modulating motor control. However, their acute effects on maximal isometric back-extensor strength in youth athletes under controlled laboratory conditions remain unclear. **Methods**: Fourteen youth golfers (15.8 ± 0.5 years) performed maximal voluntary isometric back-extension tasks under nine cueing conditions: three internal, three external, and three golf-specific. The task involved exerting maximal force against a fixed, immovable resistance while maintaining standardized trunk and hip positions to ensure consistent execution. Cueing was delivered verbally in a standardized manner across participants and sessions. Maximal isometric strength was compared across conditions using repeated-measures analyses. **Results**: Maximal isometric back-extensor strength was significantly (*p* = 0.004 η_p_^2^ = 0.34) lower with internal cues (57.1 ± 16.0 kg) compared with external (68.2 ± 13.0 kg) and golf-specific (68.1 ± 12.5 kg) cues. Specifically, the internal cues ‘engage your glutes and hamstrings’, ‘tighten your core’, and ‘maintain a neutral spine’ produced lower force than all external cues and the golf-specific cue ‘focus on using your lower body to create a stable base for your golf swing’. Among internal cues, ‘engage your glutes and hamstrings’ resulted in the lowest torque. **Conclusions**: External and certain golf-specific verbal cues acutely enhance maximal isometric back-extensor force more effectively than internal cues in a controlled laboratory setting. While these results inform how attentional focus can modulate acute force output in youth athletes, the task does not replicate the dynamic, rotational nature of the golf swing, and the findings should not be interpreted as direct indicators of golf performance. Future research should explore long-term adaptations and assess transfer to sport-specific, dynamic movements.

## 1. Introduction

The development of trunk and back extensor strength is essential for general athletic performance, postural stability, and injury prevention in youth athletes [1,2]. Strong back extensors contribute to overall force production and movement control while reducing the risk of overuse injuries such as low-back pain, which is common in both professional and youth athletes [2]. Identifying strategies that acutely enhance maximal isometric back-extensor strength is, therefore, of practical interest for coaches and practitioners aiming to support safe and effective physical development.

One factor that has received growing attention in motor control and strength research is attentional focus during movement. According to the constrained action hypothesis, directing attention internally toward specific body parts or muscles can interfere with automatic motor control, reducing efficiency [3]. In contrast, external focus cues, which direct attention toward movement outcomes or external objects, are thought to enhance force production and performance by freeing the motor system from conscious constraints [4,5]. Evidence from meta-analyses indicates that external focus generally improves strength, power, and accuracy across populations and tasks [4,5]. In maximal isometric tasks, external cues increase torque output and neuromuscular efficiency by reducing antagonist co-activation, whereas internal cues increase target muscle activation without consistently improving net force [6,7,8]. These effects may be particularly pronounced in youth athletes, whose motor control systems are still developing and who may be sensitive to subtle differences in cue phrasing.

Beyond general internal and external cues, sport-specific instructions provide a third category. By embedding task-relevant cues—such as references to swing mechanics or a stable base in golf—these instructions may enhance motor imagery and the engagement of existing motor schemas [3,9,10,11]. While the present study included a maximal isometric back-extension task in the sagittal plane—which does not replicate the dynamic, rotational, and sequential nature of a golf swing—it provides a controlled laboratory model to examine how attentional focus influences maximal force output and neuromuscular coordination under standardized conditions.

To our knowledge, no study has compared internal, external, and sport-specific verbal cues on maximal isometric back-extensor strength in youth athletes within such a controlled model. Understanding these effects has practical relevance for athletic development, as effective cueing can optimize acute motor output, training engagement, and muscle recruitment in youths, even outside of sport-specific contexts [12,13]. Accordingly, the aim of this study was to examine the acute effects of internal, external, and golf-specific verbal cues on maximal isometric back-extensor strength in youth golfers. Based on the constrained action hypothesis and existing evidence, we hypothesized that (1) external cues would elicit greater maximal torque than internal cues, and (2) golf-specific cues would match or slightly enhance the effects of general external cues due to task relevance and engagement of sport-specific motor imagery, within the limits of this controlled, non-dynamic task.

## 2. Materials and Methods

To examine any potential effects of internal cues, external cues and golf-specific cues on isometric back extensor strength performance in youth golfers, a crossover design was adopted across various conditions. Participants undertook a maximal isometric back extensor test while they were given a specific coaching cue (internal, external or golf-specific) prior to relating their performance in random order.

### 2.1. Participants

A priori power analysis was conducted using G*Power software (Version 3.1.9.4, University of Kiel, Kiel, Germany) with the F test family (ANOVA: repeated measures, within factors). The sample size calculation was based on a statistical power of 0.80, a significance level of 0.05, and an effect size of f = 0.40 (large effect) based on earlier studies that compared the effect of internal and external focus cues [4,5]. The analysis indicated that 12 kickboxers were needed to achieve 80% power.

Fourteen male youth golfers (age: 15.7 ± 0.5 yrs.; body mass: 53.5 ± 8.9 kg; height: 1.62 ± 0.08 m) from a regional golf team were enrolled in this study. The participants had undergone systematic golf training for 2.5 years prior to the study, carrying out 4–5 weekly training sessions. During the time of the study, all players competed in the top national golf division in their country of origin, and they undertook the same daily school and golf team-training schedules. Legal guardians and participants provided written informed consent and assent after a thorough explanation of the objectives and the scope of the research project, including the procedures, risks, and benefits of the study. The study adhered to the Declaration of Helsinki and was approved by the Local Clinical Research Ethics Committee (Personal Protection Committee; Code: N° 0326//2025, on 20 November 2024). Written informed consent was obtained from parents/legal representatives of all participants. Prior to baseline testing, all players were examined by a physician of the golf club to make sure that they were free from any injuries, orthopedic limitations or illnesses that might prevent them from taking part in the investigation.

### 2.2. Procedure

One week prior to data collection, participants attended a familiarization session to become accustomed to the testing procedures and the verbal cues. During this session, athletes were instructed on the proper technique for the maximal isometric back-extension test, including standardized trunk and hip positions to ensure consistent execution across trials.

Prior to testing, participants completed a ten-minute standardized warm-up, consisting of light cardiovascular activity and dynamic stretching of the upper and lower body. No performance-specific verbal cues were provided during the warm-up. Between testing trials, participants were encouraged to maintain low-intensity movement to remain prepared for subsequent maximal efforts. Body height and mass were measured on the same day using a wall-mounted stadiometer (Florham Park, NJ, USA) and an electronic scale (Baty International, West Sussex, UK), respectively.

It should be noted that the isometric back-extension task was performed in a sagittal plane, maximal-effort pull, which does not replicate the dynamic, rotational, and sequential characteristics of a golf swing. The test isolates vertical pulling force rather than transverse-plane rotational torque, and the posture was held isometrically rather than performed dynamically. While certain cues were framed in a golf-related context, the task reflects a controlled laboratory model of attentional focus and acute motor output modulation, rather than a direct assessment of golf-specific performance. This was acknowledged, and findings were interpreted as indicative of cueing effects on trunk extensor activation under standardized conditions, not as evidence of improved golf swing mechanics or sport-specific force production.

**Figure 1:**
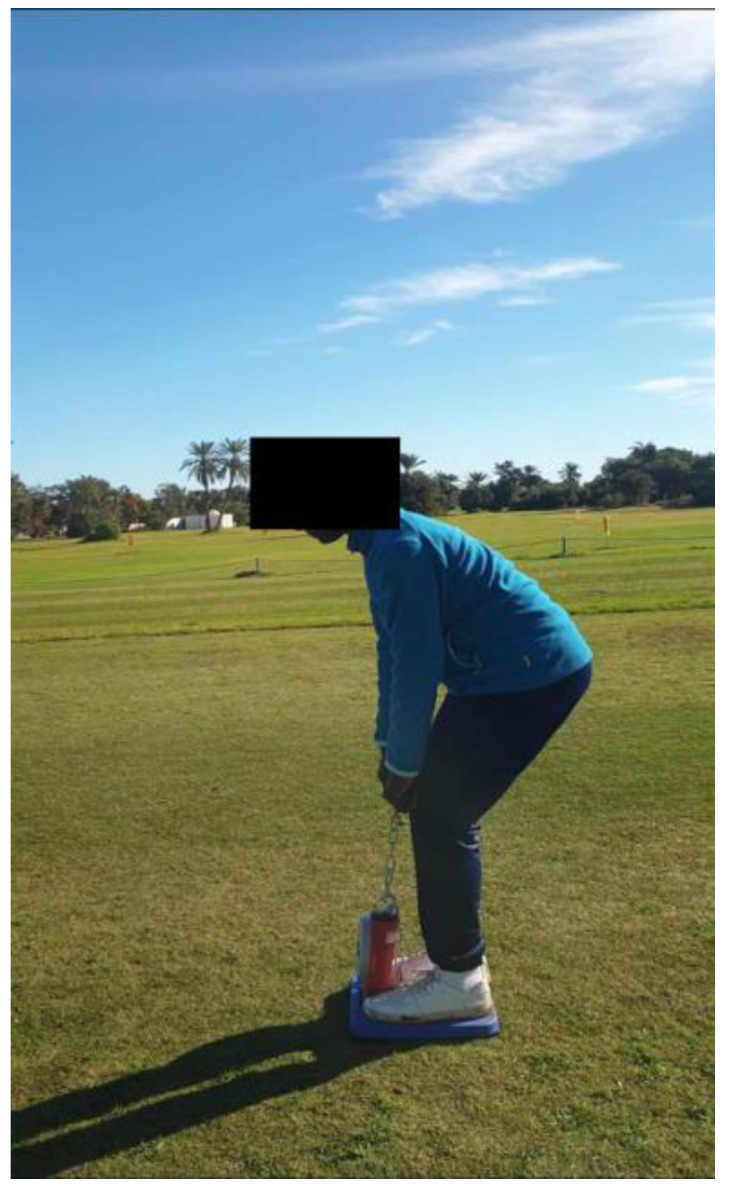
Test setup for measuring maximal isometric back extensor strength.

Maximal isometric back extensor strength was measured in kilograms using a back and leg dynamometer (Takei, Tokyo, Japan) as previously described [14,15]. All assessments were conducted in the same open-field setting (Figure 1), using an identical testing station placed on a firm, level surface. To minimize diurnal variation and environmental influences, measurements were performed at a consistent time of day (16:00–18:00 h) under comparable ambient conditions (temperature: 24–27 °C; humidity: 50–60%). Subjects stood on the dynamometer foot platform with feet shoulder-width apart and gripped the handlebars aligned across the thighs. The dynamometer chain was adjusted so that the knees were flexed at approximately 130° and the trunk was flexed at approximately 30°, positioning the bar at the patella level. The knee (≈130°) and trunk (≈30°) flexion angles were visually estimated to approximate the posture typically adopted during the golf swing, based on previously reported biomechanical data of youth and adult golfers [16,17]. This setup allows recruitment of the trunk extensors while maintaining a stable lower-body base, reflecting the combined contribution of hip, knee, and trunk musculature to force production in a position relevant to golf-specific performance.

Subjects were then instructed to straighten their backs (stand upright) without bending the knees and to exert maximal force against the dynamometer chain. Three trials per cue condition were completed, with the highest value retained for analysis. A 30 s rest interval was provided between trials. Previous studies have reported excellent test–retest reliability for this assessment in pediatric populations, with an intraclass correlation coefficient (ICC) of 0.97 and a standard error of measurement (SEM) of 1.32% [15].

A Latin square design was used to simplify randomization and minimize potential order effects, such as fatigue or learning, on participants’ performance. Each participant was randomly assigned an order scheme (1–9), which determined the sequence of instructional cues (three internal, three external, three golf-specific) prior to performing the isometric back-extensor task [18]. Testing was conducted across three separate days, with at least 48 h of rest between sessions. Each day, participants completed three different cues under various conditions according to their assigned order (Table 1), ensuring that all nine cues were tested over the three days.

Within each condition, participants performed three maximal voluntary isometric back-extension trials, separated by 2–3 min of rest. All verbal cues were delivered by the same investigator, who followed a standardized script to ensure consistent tone, duration, and timing across participants and testing sessions (2–3 s before each trial). Participants were instructed to exert maximal effort and maintain the contraction for 2–3 s following cue delivery. This procedure ensured uniform attentional focus across trials while isolating the acute effects of the different cue types on maximal force output.

The order of cueing conditions was pseudo-randomized, meaning that participants did not receive a purely random sequence but rather a controlled order designed to reduce potential sequencing effects. Specifically, a Latin square design was used to counterbalance condition order across participants, ensuring that each cueing condition appeared equally often in each position [19,20]. For analysis, the best attempt from each cue condition was retained.

The verbal cues were formulated based on established guidelines from attentional focus research [3,21], ensuring clarity, brevity, and contextual relevance to golf-specific movements. The wording and sentence structure were designed to distinctly elicit either an internal (body-oriented) or external (effect-oriented) focus of attention, consistent with prior studies demonstrating the psychometric reliability and effectiveness of similar cue formats [22].

### 2.3. Statistical Analysis

The assumption of normality was evaluated using the Shapiro–Wilk test. A repeated-measures ANOVA was conducted to assess the effect of the types of cues and the nine experimental conditions separately on the maximum isometric load. Mauchly’s test of sphericity was applied to assess the assumption of sphericity. When this assumption was violated, Greenhouse–Geisser corrections were applied to the degrees of freedom. Effect sizes were reported using partial eta squared (η_p_^2^). Effect size was evaluated with η_p_^2^ (ETA squared), where 0.01 < η_p_^2^ < 0.06 constitutes a small effect, 0.06 < η_p_^2^ < 0.14 constitutes a medium effect, and η_p_^2^ > 0.14 constitutes a large effect [23]. Post hoc pairwise comparisons were adjusted using the Holm–Bonferroni correction to control for Type I errors. Data are presented as mean ± standard deviation. All analyses were conducted in JASP (version 0.18.1, Amsterdam, The Netherlands). Statistical significance was set at *p* < 0.05.

## 3. Results

**Figure 2:**
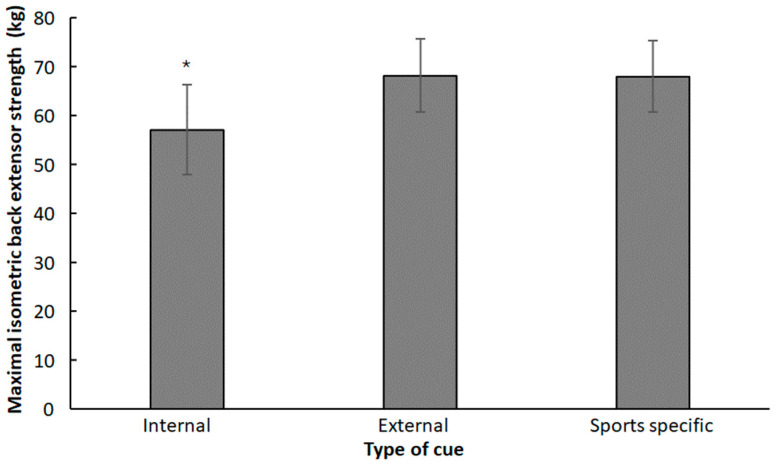
Mean (95% confidence intervals) maximal isometric back extensor strength per type of cue. * indicates significant differences with the other two types of cues.

A significant difference between the types of cues was found on maximal isometric back extensor strength with a large effect (F_1.2,15.67_ = 6.83, *p* = 0.004, η_p_^2^ = 0.34). Post hoc comparisons revealed that maximal isometric back extensor strength was significantly lower when using internal cues (~11.0 kg, 16.2%) compared to the other two types of cues (Figure 2).

**Figure 3:**
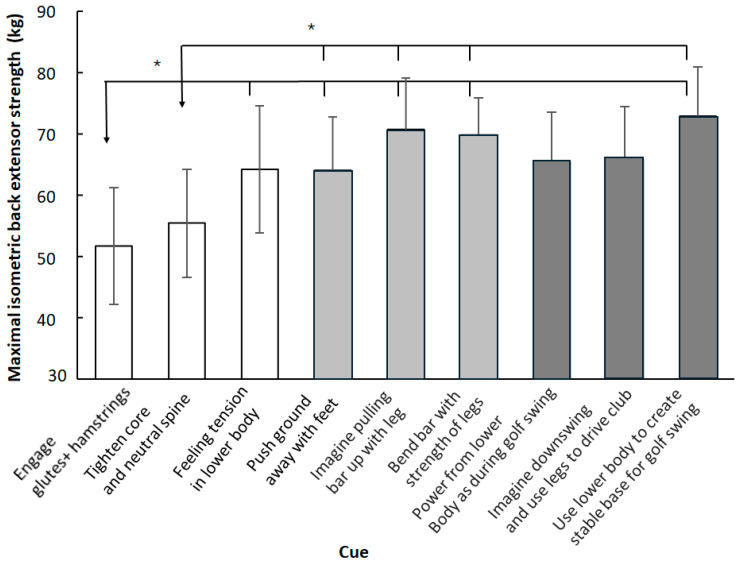
Mean (95% confidence intervals) maximal isometric back extensor strength for each cue. * indicates significantly lower maximal isometric back strength between this cue and the others at a *p* < 0.05 level.

When analyzing each cue, a significant effect was observed (F_2.2,28.2_ = 6.89, *p* < 0.001, η_p_^2^ = 0.35). Post hoc comparisons revealed that the internal cues of ‘engage your glutes and hamstrings’ and ‘tighten your core and maintain a neutral spine’ resulted in significantly lower maximal isometric back extensor force output compared with all three external focus cues and with the cue to ‘focus on using your lower body to create a stable base for your golf swing’. In addition, performance was significantly lower with the ‘engage your glutes and hamstrings’ cue compared with the other internal cue stating ‘focus on feeling the tension in your lower body muscles’ (Figure 3).

## 4. Discussion

The present study examined the acute effects of internal, external, and golf-specific verbal cues on maximal isometric back extensor force output in youth golfers. The main finding was that external and golf-specific cues elicited higher maximal force with a large effect size compared with internal cues. Specifically, the internal cues ‘engage your glutes and hamstrings’ and ‘tighten your core and maintain a neutral spine’ produced significantly lower force than all external cues and the golf-specific cue ‘focus on using your lower body to create a stable base for your golf swing’. Among internal cues, ‘engage your glutes and hamstrings’ resulted in the lowest torque. Overall, these results provide insight into how attentional focus affects neuromuscular recruitment in youth athletes, highlighting that externally directed and carefully designed sport-specific cues can acutely enhance force output.

These findings align with the constrained action hypothesis [3], which proposes that directing attention inward toward the body or specific muscles can disrupt automatic motor processes and reduce net force production. For instance, internal cues such as “engage your glutes and hamstrings” may increase activation of target muscles but can also lead to unnecessary co-contraction or interfere with inter-muscular coordination [7]. In contrast, external and sport-specific cues—such as “push the ground away” or “focus on using your lower body to create a stable base for your golf swing”—encourage attention toward movement outcomes or familiar motor imagery, which may improve coordination and allow greater net force production. These examples illustrate how the phrasing and focus of verbal cues can meaningfully alter acute performance.

It is important to note that the maximal isometric back-extension task performed in the sagittal plane does not replicate the dynamic, rotational, and sequential nature of a golf swing. In contrast to the swing, which primarily involves transverse-plane trunk rotation and coordinated segmental sequencing, the back-extension movement is not a primary component of golf performance. Moreover, the isometric, maximal-effort setup differs from how golfers typically generate force during play, where movements are brief, rotational, and modulated by shot type. Thus, while golf-specific cues were included, their observed effects reflect acute modulation of isometric force under controlled laboratory conditions rather than improvements in swing performance.

Variability in cue effectiveness highlights the influence of precise wording, type of motor imagery, and individual interpretation. For example, cues emphasizing body sensation (“feel the tension in your lower body”) were less effective than outcome-oriented instructions (“push the ground away”), suggesting that attentional allocation toward the task goal enhances performance. This underscores a methodological challenge in cueing research: subtle differences in phrasing, prior exposure to cues, individual attentional capacity, or participant motivation may substantially influence outcomes [24,25,26]. Future studies should standardize cue formulation, account for prior experience with cues, and consider attentional capacity as a potential confounding variable to improve reproducibility.

The adolescent period is characterized by rapid neuromuscular development, and youth athletes may be particularly sensitive to attentional focus strategies. The pronounced differences between internal and external/golf-specific cues observed here suggest that verbal instruction can meaningfully influence motor output during development, with implications for motor learning, engagement, and injury prevention [27,28,29]. Practically, coaches should tailor cue delivery to the individual athlete, emphasizing clear, externally focused instructions or sport-specific imagery. Effective examples include “drive through your legs to generate force” or “imagine transferring power from your lower body to the club,” which guide attention toward outcomes while remaining contextually relevant.

Several limitations should be considered. First, this study only assessed the acute effects of verbal cueing on maximal isometric back-extensor force, so the impact of repeated or prolonged exposure on trunk strength, motor coordination, or injury risk remains unknown. Longitudinal studies are needed to determine whether the acute benefits of external and certain sport-specific cues translate into chronic performance improvements or enhanced neuromuscular control. Second, the sample was small and homogeneous (*n* = 14), limiting generalizability to older, more experienced, or elite golfers, as well as athletes in other sports or age groups. Third, the study did not include direct assessment of golf-specific performance; the sagittal-plane back-extension task isolates vertical pulling force rather than rotational torque, so its ecological validity for swing mechanics, drive distance, or shot-specific adaptations is limited. Fourth, participant factors such as prior exposure to verbal cues, individual attentional capacity, and motivation could have influenced outcomes. Future research should incorporate ecologically valid, sport-specific assessments such as rotational trunk torque testing, cable-resisted or medicine-ball rotational tasks, and monitor golf swings to better examine both the acute and chronic effects of attentional focus strategies on golf-specific performance. Standardization and psychometric validation of cues will be critical for reproducibility and to clarify underlying neuromuscular mechanisms.

## 5. Conclusions

This study demonstrated that verbal cueing can acutely influence maximal isometric back extensor strength in youth golfers. Overall, external and certain golf-specific cues tended to elicit higher force outputs than internal, body-focused instructions, supporting attentional focus and motor learning theories, which suggest that directing attention toward movement outcomes or task-relevant imagery enhances neuromuscular efficiency. However, not all sport-specific cues were consistently advantageous, as some produced effects similar to internal instructions.

From a practical standpoint, practitioners should recognize that external and carefully selected sport-specific cues can support greater back strength expression during testing, warm-up routines, or conditioning sessions, but their effectiveness may vary with cue phrasing, individual interpretation, and prior experience. Methodological considerations, including the small sample size and potential differences in participants’ exposure to cues or attentional capacity, limit generalizability.

Future research should examine the long-term impact of cueing strategies on strength development, motor learning, golf-specific performance, and injury prevention, while considering age and developmental differences. The use of advanced biomechanical tools (e.g., high-density EMG, motion capture, force plates) could further clarify the neuromuscular mechanisms underlying these effects and inform evidence-based coaching practices.

## Figures and Tables

**Table 1:** Instructions on internal, external, and golf-specific cues were provided to the study participants.

Cue Type	Instructions
**Internal cues**	1. Engage your glutes and hamstrings 2. Tighten your core and maintain a neutral spine 3. Focus on feeling the tension in your lower body muscles
**External cues**	1. Push the ground away with your feet 2. Imagine pulling the bar up with your legs 3. Try to make the bar bend with the strength of your legs
**Golf-specific cues**	1. Generate power from your lower body as you would during a golf swing 2. Imagine you’re starting your downswing and using your legs to drive the club forward 3. Focus on using your lower body to create a stable base for your golf swing

## Data Availability

The original contributions presented in this study are included in the article. Further inquiries can be directed to the corresponding author.

## References

[1] Cole M.H., Grimshaw P.N. (2016). The biomechanics of the modern golf swing: Implications for lower back injuries. Sports Med..

[2] Smith J.A., Hawkins A., Grant-Beuttler M., Beuttler R., Lee S.-P. (2018). Risk factors associated with low back pain in golfers: A systematic review and meta-analysis. Sports Health.

[3] Wulf G., Lewthwaite R. (2016). Optimizing performance through intrinsic motivation and attention for learning: The OPTIMAL theory of motor learning. Psychon. Bull. Rev..

[4] Bull H.G., Atack A.C., North J.S., Murphy C.P. (2023). The effect of attentional focus instructions on performance and technique in a complex open skill. Eur. J. Sport Sci..

[5] Nevo M., Halperin I., Ziv G. (2024). Do the effects last? A comparison between internal and external focus of attention instructions on golf putting accuracy over multiple days. PeerJ.

[6] Neumann D.L. (2019). A systematic review of attentional focus strategies in weightlifting. Front. Sports Act. Living.

[7] Wiseman S., Alizadeh S., Halperin I., Lahouti B., Snow N.J., Power K.E., Button D.C. (2020). Neuromuscular mechanisms underlying changes in force production during an attentional focus task. Brain Sci..

[8] Lohse K.R., Sherwood D.E., Healy A.F. (2011). Neuromuscular effects of shifting the focus of attention in a simple force production task. J. Mot. Behav..

[9] Shafizadeh M., McMorri T., Sproule J. (2011). Effect of different external attention of focus instruction on learning of golf putting skill. Percept. Mot. Ski..

[10] An J., Wulf G. (2024). Golf skill learning: An external focus of attention enhances performance and motivation. Psychol. Sport Exerc..

[11] Wulf G., Lauterbach B., Toole T. (1999). The learning advantages of an external focus of attention in golf. Res. Q. Exerc. Sport.

[12] Lloyd R.S., Oliver J.L., Radnor J.M., Rhodes B.C., Faigenbaum A.D., Myer G.D. (2015). Relationships between functional movement screen scores, maturation and physical performance in young soccer players. J. Sports Sci..

[13] Wachholz F., Tiribello F., Mohr M., van Andel S., Federolf P. (2020). Adolescent awkwardness: Alterations in temporal control characteristics of posture with maturation and the relation to movement exploration. Brain Sci..

[14] Koley S., Jha S., Sandhu J.S. (2012). Study of back strength and its association with selected anthropometric and physical fitness variables in inter-university hockey players. Anthropologist.

[15] Hammami R., Chaouachi A., Makhlouf I., Granacher U., Behm D.G. (2016). Associations between balance and muscle strength, power performance in male youth athletes of different maturity status. Pediatr. Exerc. Sci..

[16] Kim S.E., Pham N.S., Park J.H., Ladd A., Lee J. (2022). Potential biomechanical risk factors on developing lead knee osteoarthritis in the golf swing. Sci. Rep..

[17] Lin Z.-J., Peng Y.-C., Yang C.-J., Hsu C.-Y., Hamill J., Tang W.-T. (2023). Lower limb biomechanics during the golf downswing in individuals with and without a history of knee joint injury. Bioengineering.

[18] Moran J., Hammami R., Butson J., Allen M., Mahmoudi A., Vali N., Lewis I., Samuel P., Davies M., Earle J. (2023). Do verbal coaching cues and analogies affect motor skill performance in youth populations?. PLoS ONE.

[19] Wickens T.D., Keppel G. (2004). Design and Analysis: A Researcher’s Handbook.

[20] Bradley J.V. (1958). Complete counterbalancing of immediate sequential effects in a Latin square design. J. Am. Stat. Assoc..

[21] Wulf G. (2013). Attentional focus and motor learning: A review of 15 years. Int. Rev. Sport Exerc. Psychol..

[22] Marchant D.C., Greig M., Scott C. (2009). Attentional focusing instructions influence force production and muscular activity during isokinetic elbow flexions. J. Strength Cond. Res..

[23] Cohen J. (1988). Statistical Power Analysis for the Behavioral Sciences.

[24] Halperin I., Williams K.J., Martin D.T., Chapman D.W. (2016). The effects of attentional focusing instructions on force production during the isometric midthigh pull. J. Strength Cond. Res..

[25] Grgic J., Mikulic P. (2021). Effects of attentional focus on muscular endurance: A meta-analysis. Int. J. Environ. Res. Public Health.

[26] Wu W.F., Porter J.M., Brown L.E. (2012). Effect of attentional focus strategies on peak force and performance in the standing long jump. J. Strength Cond. Res..

[27] Noroozi T., Saemi E., Doustan M., Singh H., Aiken C.A. (2024). The effect of internal, external, and holistic focus of attention on standing long jump performance in novice and skilled karatekas. Eur. J. Sport Sci..

[28] Barillas S.R., Oliver J.L., Lloyd R.S., Pedley J.S. (2021). Cueing the youth athlete during strength and conditioning: A review and practical application. Strength Cond. J..

[29] Siltanen S., Bottas R. (2022). Instructions for external focus of attention improved taekwondo kicking performance only among less skilled youth. Percept. Mot. Ski..

